# The performance of tongue swabs for detection of pulmonary tuberculosis

**DOI:** 10.3389/fcimb.2023.1186191

**Published:** 2023-09-06

**Authors:** Christopher S. Ealand, Astika Sewcharran, Julian S. Peters, Bhavna G. Gordhan, Mireille Kamariza, Carolyn R. Bertozzi, Ziyaad Waja, Neil A. Martinson, Bavesh D. Kana

**Affiliations:** ^1^ Department of Science and Innovation/National Research Foundation Centre of Excellence for Biomedical TB Research, School of Pathology, Faculty of Health Sciences, University of the Witwatersrand and the National Health Laboratory Service, Johannesburg, South Africa; ^2^ Department of Biology, Stanford University, Stanford, CA, United States; ^3^ Department of Chemistry, University of California, Berkeley, Berkeley, CA, United States; ^4^ Department of Chemistry, Stanford University, Stanford, CA, United States; ^5^ Howard Hughes Medical Institute, Stanford University, Stanford, CA, United States; ^6^ Perinatal HIV Research Unit (PHRU), University of the Witwatersrand, Johannesburg, South Africa; ^7^ Johns Hopkins University, Centre for Tuberculosis Research, Baltimore, MD, United States

**Keywords:** differentially culturable tubercle bacilli (DCTB), tuberculosis, tongue swabs, *Mycobacterium tuberculosis*, MPN (most probable number)

## Abstract

**Introduction:**

Oral and/or tongue swabs have demonstrated ability to detect *Mycobacterium tuberculosis (Mtb)* in adults with pulmonary tuberculosis (TB). Swabs provide useful alternative specimens for diagnosis of TB using molecular assays however, the diagnostic pickup by culture requires further improvement and development. Several studies identified the presence of differentially culturable tubercle bacilli (DCTB) populations in a variety of clinical specimens. These organisms do not grow in routine laboratory media and require growth factors in the form of culture filtrate (CF) from logarithmic phase cultures of *Mtb* H37Rv.

**Methods:**

Herein, we compared the diagnostic performance of sputum and tongue swabs using Mycobacterial Growth Indicator Tube (MGIT) assays, Auramine smear, GeneXpert and DCTB assays supplemented with or without CF.

**Results:**

From 89 eligible participants, 83 (93%), 66 (74%) and 79 (89%) were sputum positive by MGIT, smear and GeneXpert, respectively. The corresponding tongue swabs displayed a lower sensitivity with 39 (44%), 2 (2.0%) and 18 (20%) participants respectively for the same tests. We aimed to improve the diagnostic yield by utilizing DCTB assays. Sputum samples were associated with a higher positivity rate for CF-augmented DCTB at 82/89 (92%) relative to tongue swabs at 36/89 (40%). Similarly, sputum samples had a higher positivity rate for DCTB populations that were CF-independent at 64/89 (72%) relative to tongue swabs at 26/89 (29%). DCTB positivity increased significantly, relative to MGIT culture, for tongue swabs taken from HIV-positive participants. We next tested whether the use of an alternative smear stain, DMN-Trehalose, would improve diagnostic yield but noted no substantial increase.

**Discussion:**

Collectively, our data show that while tongue swabs yield lower bacterial numbers for diagnostic testing, the use of growth supplementation may improve detection of TB particularly in HIV-positive people but this requires further interrogation in larger studies.

## Introduction

Historically, TB diagnosis has relied on symptomatic people reporting to health care facilities for diagnostic testing which typically involves microbiological or molecular-based (i.e. nucleic acid) approaches to confirm infection (Ho et al., 2016). Sputum is routinely used as the clinical specimen of choice but can be difficult to produce, or yields equivocal results, in certain vulnerable populations such as children or people living with HIV (PLWH). Moreover, without adequate safety measures, aerosol production during coughing, or respiratory maneuvers, that occur during sample collection can be hazardous to healthcare workers and other patients (Andama et al., 2022). Alternative specimen types which are non-invasive, safer and easy to collect are required. Recently, the use of oral/tongue swabs has gained traction as alternative specimens for TB testing, albeit with a wide range of reported sensitivities and specificities (Wood et al., 2015; Luabeya et al., 2019; Mesman et al., 2019; Nicol et al., 2019; Deviaene et al., 2020; Lima et al., 2020; Mesman et al., 2020; Molina-Moya et al., 2020; Song et al., 2021; Wood et al., 2021; Abdulgader et al., 2022; Cox et al., 2022a; LaCourse et al., 2022). Culture remains a gold standard for TB diagnosis, therefore developing sample collection and laboratory processes that optimize a culture-based diagnostic yield from oral swabs will be required to show proof of concept of this approach.

Recent studies, including those from our group, have demonstrated the presence of non-replicating, drug-tolerant, differentially culturable tubercle bacteria (DCTB) in the sputum of participants with active pulmonary or extra-pulmonary TB (Chengalroyen et al., 2016; Rosser et al., 2018; Almeida Júnior et al., 2020; Gordhan et al., 2021; Mesman et al., 2021; Zainabadi et al., 2021; Glenn et al., 2022; Peters et al., 2022). These bacteria are unable to grow on solid media and only emerge following liquid culture with growth factor supplementation. Supplementation is typically in the form of culture filtrate (CF), derived from logarithmic phase *Mtb*, which is mixed with fresh media to serve as the growth media for sputum samples. It is hypothesized that as bacteria grow in culture, they secrete growth factors that enable proliferation of the whole population. In a logarithmic phase culture of *Mtb*, once the bacteria are removed, the resulting CF will contain these growth factors. Supplementation of growth media with this CF could promote the growth of differentially culturable bacteria (Mukamolova et al., 2010; Chengalroyen et al., 2016). Recovery of DCTB can also be enhanced with lipid rich media (Mesman et al., 2021). Growth assays, in the form of liquid limiting dilutions (LLDs), entail adding limiting dilutions of the sputum into media with or without (CF^+^ or CF^-^) to determine bacterial counts *via* turbidity. These assays yield the Most Probable Number (MPN) of bacteria present in the sample. Our previous work demonstrated that application of DCTB assays on sputum allowed for detection of individuals missed by routine culture (Chengalroyen et al., 2016; McIvor et al., 2021). In addition, it has been shown that approximately 90% of the bacilli in sputum are persisters that can grow in liquid without the need for growth supplementation, but not on solid plates (Dhillon et al., 2014). It remains unclear what factors drive bacteria into the DCTB state but recent evidence indicates that oxidative stress plays a significant role in this regard (Saito et al., 2021). We hypothesized that since saliva plays a vital role in defense against various microbial species in general (Salvatori et al., 2016; Zhang et al., 2016), anti-bacterial compounds such as hydrogen peroxide, lactoferrin and lysozymes may drive *Mtb* bacilli residing in the oral cavity into the DCTB state, thereby reducing routine culture yield.

In addition to MPN culture-based approaches to detect *Mtb*, recent studies have highlighted the use of novel probes to detect viable organisms in sputa. Microscopy-based diagnostics of acid-fast stained sputum are typical in low-resource settings due to low cost and fast turnaround times. Stains such as Auramine and Ziehl-Neelsen are clinical standards but are unable to distinguish between viable and dead bacteria. Moreover, sensitivities can range between 20 to greater than 80% (Ryan et al., 2014). Fluorogenic probes such as DMN-Trehalose leverage the substrate promiscuity of the antigen 85 (Ag85) complex that catalyzes mycolyation of trehalose to form trehalose monomycolates (TMMs) (Belisle et al., 1997). The solvatochromic nature of this probe causes it to only ‘turn on’ or fluoresce following incorporation into the mycobacterial cell wall (Kamariza et al., 2018).

In this study, we interrogated the diagnostic utility of tongue swabs, relative to sputum, in individuals with confirmed TB (with or without HIV co-infection). Several clinical tests were utilized in this regard, including MGIT, auramine smear microscopy, GeneXpert and DCTB assays in a well characterized prospective clinical cohort from South Africa (Soweto).

## Results

### Study design and population

**Figure 1:**
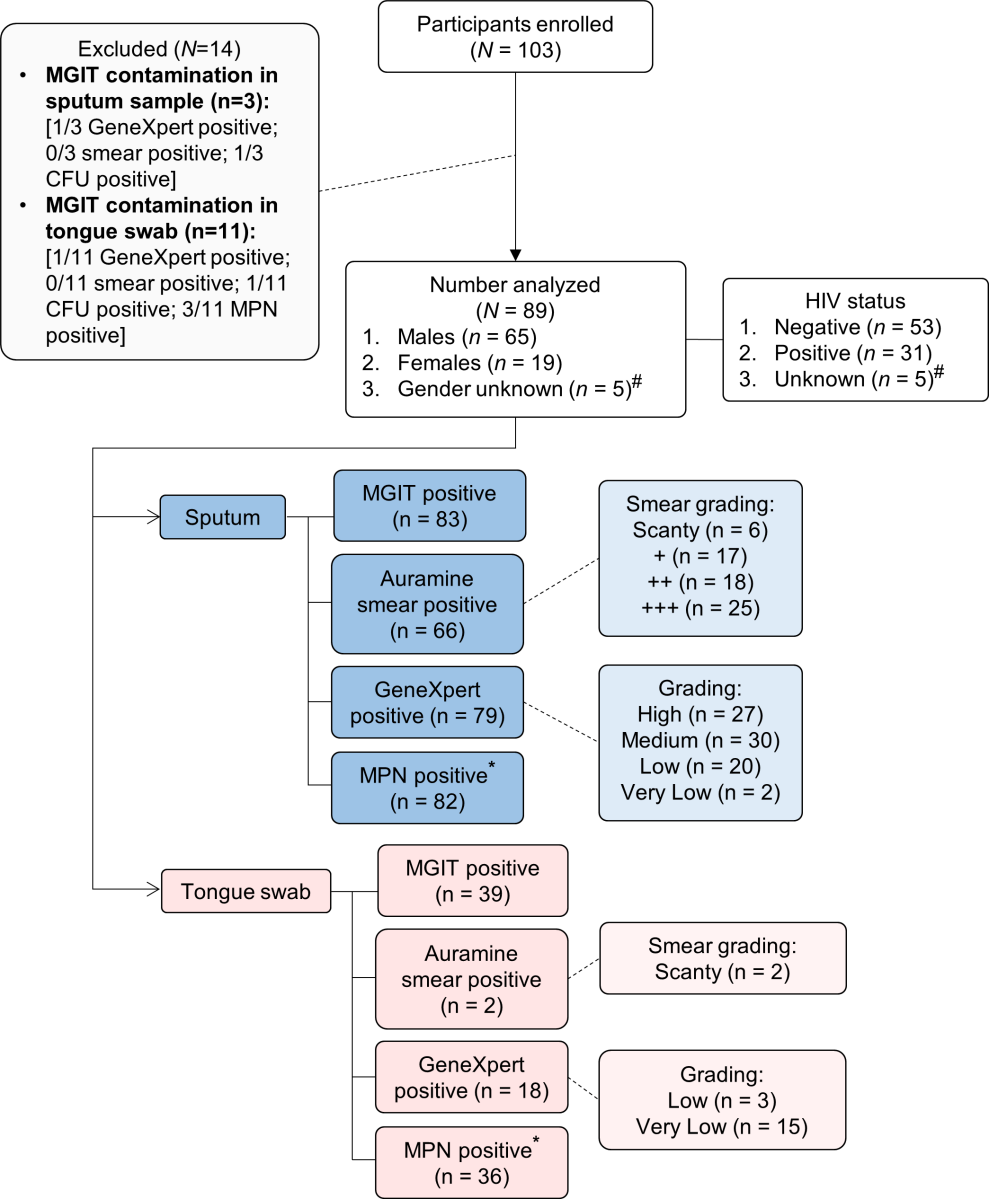
Strobe diagram highlighting cross-sectional study comparing sputum and tongue swabs as specimens for TB diagnostic testing. A total of 103 participants were analyzed in this study. Participants were recruited based on a strong clinical indication of TB disease either *via* an Auramine positive smear or positive GeneXpert result. Of these, 14 were excluded due to contaminated MGIT cultures for either the sputum and/or tongue swab. For the 89 participants enrolled, two specimen types were collected for analysis (sputum and tongue swab). For the sputum samples, 83 were MGIT-positive, 66 were smear-positive, 79 were GeneXpert-positive and 82 contained DCTB. In the corresponding tongue swabs, 39 were MGIT-positive, 2 were smear-positive, 18 were GeneXpert-positive and 36 contained DCTB. #Gender and HIV status were not available. *Corresponds to CF-augmented MPN assays only. Definitions: MGIT (Mycobacterial Growth Indicator Tube); CFU (Colony Forming Units); MPN (Most Probable Number); DCTB (Differentially Culturable Tubercle Bacilli).

A total of 103 participants with TB were enrolled in this study (**Figure 1**). These participants were recruited prospectively from primary healthcare settings in Soweto based on either a positive GeneXpert test or auramine smear. The gender and HIV status for 5/89 participants (5.6%) were not captured but of those with data, 65/89 (73%) were male and 19/89 (21%) were female. There were 53/89 (60%) and 31/89 (35%) HIV-negative and HIV-positive cases, respectively (**Table 1**). Of the 31 HIV-positive participants, 10 (32%) were on anti-retroviral therapy (ART) and CD4 counts were available for 16; the median was 197.5 cells/mm^3^. All sputum samples were classified as ‘rifampicin sensitive’ on the GeneXpert whereas one tongue swab from one participant was discordant (RIF-resistant). Two HIV-positive participants had diabetes. Overall, BMIs could be calculated for 64 participants with a median of 19.77 units. When stratified by HIV status, HIV-negative participants (n = 40) had a higher median BMI (20.31) relative to HIV-positive participants (n = 24) at 19.18. Our exclusion criteria allowed for multidrug TB treatment for up to 5 days. Out of the 89 participants recruited, 36 (40%) indicated “yes” to being on drug treatment prior to enrollment; 33 (37%) indicated “no” and data was not captured for the remaining 20 (22%) (**Table 1**). Of the participants that were on treatment, 7/36 (19%) received medication for up to 3 days, 3/36 (8%) received medication for 1 day and the remainder, 26/36 (72%), either received medication on the same day or no time-frame was indicated. Despite this, bacterial loads retrieved from sputum samples appeared not to be significantly affected as a result of treatment. Specifically, 35/36 (97%) were MGIT positive; 29/36 (81%) were smear positive; 35/36 (97%) were GX positive; and 33/35 (94%) positive for DCTB. As MGIT culture remains the current gold standard for TB diagnosis, fourteen participants with a contaminated MGIT for either sputum or tongue swab were excluded from further analysis. Negative cultures in either sputum or tongue swabs were included. In total, sputum and tongue swabs from 89/103 (86%) participants are therefore included in this analysis.

**Table 1:** Demographics, microbiology and diagnostic data for participants, stratified by HIV-1 infection status.

Variable		HIV-status		
	Overall (n = 89)	HIV-negative (n = 53)	HIV-positive (n = 31)	HIV unknown (n = 5)
Demographics				
Sex				
Male, n (%)	65 (73)	43 (66)	22 (34)	Unknown
Female, n (%)	19 (21)	10 (53)	9 (47)	Unknown
Age, yr, median (IQR)	34 (27 – 41)	33 (26.0 – 39.0)	35 (30.0 – 42.0)	31 (23.0 – 38.5)
Diabetes, n	2	2	0	0
On ART, n	10	N/A	10	N/A
CD4 Count, median (IQR), (n = 16)	197.5 (13.0 – 855.0)	N/A	197.5 (13.0 – 855.0)	N/A
	***Overall (n = 64)***	***HIV Negative (n = 40)***	***HIV Positive (n = 24)***	***HIV unknown (n = 0)***
BMI, median (IQR) ∞	19.77 (12.74 – 30.78)	20.31 (12.74 – 29.20)	19.18 (14.88 – 30.78)	N/A
On multi-drug anti-TB medication before enrollment				
* Yes, n (%)*	*36 (40)*	*22 (42)*	*14 (45)*	*0 (0)*
*No, n (%)*	*33 (37)*	*22 (41)*	*11 (36)*	*0 (0)*
* Unknown or not captured, n (%)*	*20 (23)*	*9 (17)*	*6 (19)*	*5 (100%)*
Sputum				
Conventional TB diagnosis, n (%)				
Smear grade negative	23 (*26*)	10 (*19*)	12 (39)	1 (20)
Smear grade positive ‡	66 (*74*)	43 (*81*)	19 (61)	4 (80)
* Scanty/+ [n (% of positive)]*	*23 (35)*	*12 (28)*	*10 (53)*	*1 (25)*
* ++*	*18 (27)*	*12 (28)*	*6 (32)*	*0*
* +++*	*25 (38)*	*19 (44)*	*3 (16)*	*3 (75)*
MGIT positive, n (*%*)	83 (93)	48 (91)	31 (*100*)	4 (80)
* Time to positivity, hrs, median (IQR)*	172 (129.0 – 256.0)*	*167.5 (131.5 – 239.3)*	*216.0 (115.0 – 267.0)*	*139.0 (108.3 – 166.8)*
GeneXpert positive, n (%)	79 (*89*)	46 (87)	29 (94)	4 (80)
* High, n (% of positive)*	*27 (34)*	*21 (46)*	*3 (10)*	*3 (75)*
* Medium, n (%)*	*30 (38)*	*17 (37)*	*12 (41)*	*1 (25)*
* Low and Very Low, n (%)*	*22 (28)*	*8 (17)*	*14 (48)*	*0 (0)*
* Cycle threshold value, median (IQR)*	*19.80 (14.59 – 23.65)*	*17.96 (15.92 – 25.04)*	*22.96 (19.57 – 26.28)*	*15.99 (15.55 – 18.06)*
MPN (bacterial load)				
MPN positive (CF-augmented), n (%)	82 (92)	49 (92)	29 (94)	4 (80)
MPN positive (CF-independent), n (%)	64 (72)	38 (72)	22 (71)	4 (80)
* CFU/ml, Log median (IQR)*	*3.56 (0.0 – 5.12)*	*3.63 (0.0 – 5.22)^#^*	*3.140 (0.0 – 5.10)*	*5.74 (2.27 – 5.99)*
* CF-dependent MPN, Log median (IQR)*	*4.26 (3.26 – 5.66)*	*4.66 (3.48 – 5.93)*	*3.93 (2.66 – 4.93)^$^*	*5.93 (2.33 – 6.60)*
* CF-independent MPN [media], Log median (IQR)*	*1.66 (0.0 – 3.460)*	*1.66 (0.0 – 3.10)*	*1.68 (0.0 – 2.93)^^^*	*5.18 (0.81 – 6.06)*
Tongue swabs				
Conventional TB diagnosis, n (%)				
Smear grade negative	87 (98)	51 (59)	31 (100)	4 (80)
Smear grade positive ‡	2 (2)	2 (67)	0	1 (20)
* Scanty/+ [n (% of positive)]*	*2 (100)*	*2 (75)*	*0*	*1 (100)*
* ++*	*0*	*0*	*0*	*0*
* +++*	*0*	*0*	*0*	*0*
MGIT positive, n (%)	39 (44)^¥^	26 (67)	9 (29)	4 (80)
* Time to positivity, hrs, median (IQR)*	*366 (306.0 – 455.0)*	*377.5 (306.8 – 468.5)*	*358.0 (294.0 – 446.0)*	*320.5 (264.0 – 358.3)*
GeneXpert positive, n (%)	18 (20)	10 (53)	6 (19)	3 (60)
* High, n (% of positive)*	*0*	*0*	*0*	*0*
* Medium, n (%)*	*0*	*0*	*0*	*0*
* Low and Very Low, n (%)*	*18 (100)*	*10 (52.6)*	*6 (100)*	*3 (100)*
* Cycle threshold value, median (IQR)*	*31.07 (29.68 – 32.13)*	*30.7 (29.61 – 32.09)*	*32.2 (31.2 – 32.9)*	*26.1 (26.0 – 29.7)*
MPN (bacterial load)				
MPN positive (CF-augmented), n (%)	36 (40)	18 (34)	17 (55)	1 (20)
MPN positive (CF-independent), n (%)	26 (29)	9 (17)	15 (48)	2 (40)
* CFU/ml, Log median (IQR)*	*0 (0 – 0)^#^*	*0 (0 – 0)*	*0 (0 – 0)*	*0 (0 – 0)*
* CF-dependent MPN, Log median (IQR)*	*0 (0 – 1.66)*	*0 (0 – 1.62)*	*0.85 (0 – 1.93)^^^*	*0 (0 – 0.59)*
* CF-independent MPN [media], Log median (IQR)*	*0 (0 – 1.02)^$^*	*0 (0 – 0)^^^*	*0 (0 – 1.93)*	*0 (0 – 0.85)*

MGIT, mycobacterial growth indicator tube; CFU/ml, colony forming units/ml; CF, culture filtrate.

∞ indicates that BMI was calculated on a subset of participants (shown above the value) for which clinical data was captured; ‡ Includes scanty, +, ++, and +++; * 86/89 MGIT positive samples; (in Sputum section) # plates of 3 samples were contaminated; $ and ^ one MPN plate contaminated; (in Tongue swabs section) ¥ 1/89 MGIT samples not done; # plates of 2 samples were contaminated; $ and ^ one MPN plate contaminated. Data are from sputum and tongue swab samples at baseline.

N/A, not applicable.

### Diagnostic performance of tongue swabs using established clinical assays

**Figure 2:**
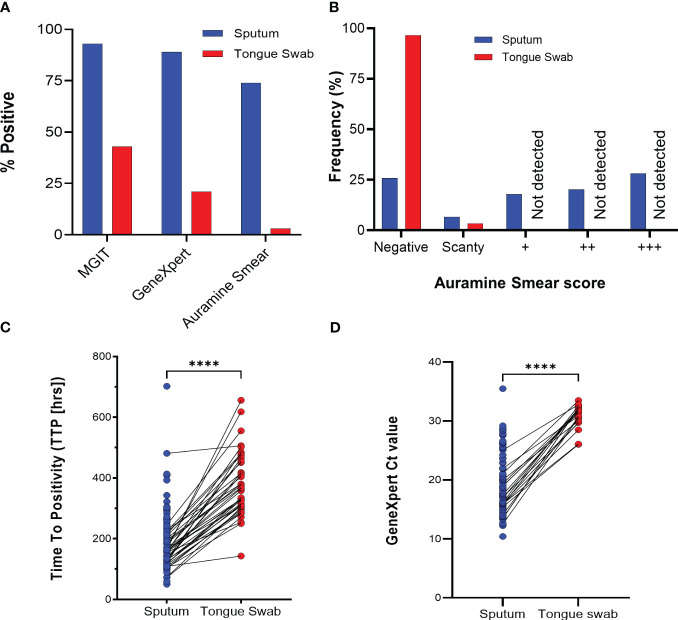
Routine tuberculosis diagnostics comparing sputum and tongue swab samples. **(A)** Relative performance of sputum and tongue swabs in detecting *Mycobacterium tuberculosis* as a function of total number of participants (N = 89). **(B)** Diagnostic yield by smear status graded according to WHO guidelines (scanty, +, ++ or +++). **(C)** Scatter plot comparing the MGIT time to positivity (TTP) in hours for positive *Mtb* in sputum and tongue swabs. Lines between points represent a positive MGIT in sputum and tongue swab for the same participant. Where no growth was detected after 42 days, a ‘negative’ was assigned but not used in the graph. The median TTP was 172 and 366 hrs for sputum and tongue swabs, respectively (**** = P<0.0001). **(D)** GeneXpert Ct values for sputum and tongue swab samples. No Ct value was interpreted as a negative result and was not used in the graph. The median Ct value was 19.16 and 31.34 for sputum and tongue swabs, respectively (**** = P<0.0001). MGIT, Mycobacterial Growth Indicator Tube; CF, culture filtrate.

The diagnostic yield from tongue swabs was compared to each corresponding sputum sample taken the same day at baseline visit. Of 89 sputum specimens, the routine clinical tests (MGIT, GeneXpert and Auramine smear) were sputum-positive for *Mtb* in 83/89 (93%), 79/89 (89%) respectively and for acid-fast bacilli (AFB) in 66/89 (74%) (**Figure 2A**); the proportions positive for *Mtb* in tongue swabs was lower at 39/89 (44%), 18/89 (20%), respectively and 2/89 (2%) were AFB positive (**Table 1**). There was participant whose tongue swab was positive and the corresponding sputum sample was scored as negative but this was due to contamination. Discordant tongue swab results – negative on the tongue in the face of a positive sputum – were more likely in those whose sputum assays suggested low bacillary loads (**Figure 2B**). Relative to sputum, tongue swabs yielded a higher median MGIT time to positivity (TTP) (366 vs 172 hours; *P*<0.0001) (**Figure 2C**) and a higher GeneXpert Ct value (31.07 vs 19.80; *P*<0.0001) (**Figure 2D**). Moreover, GeneXpert positive sputum samples displayed a range of cycle threshold (CT) values between ‘High’ (27/79 or 34%), ‘Medium’ (30/79 or 40%), ‘Low’/’Very Low’ (22/79 or 28%) whereas GeneXpert CT values for positive tongue swabs were all (18/18 or 100%) classified as ‘Low’/’Very Low’ (**Table 1**).

### Detection of *Mtb* using DMN-Tre

**Figure 3:**
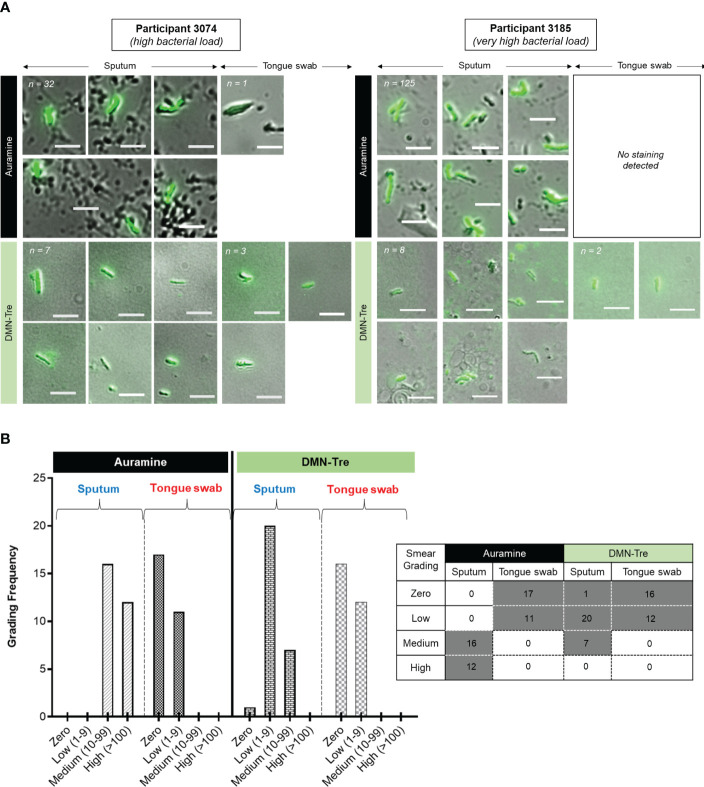
Comparison of Auramine and DMN-Tre staining on sputum and tongue swab samples. **(A)** Two representative samples showing Auramine and DMN-Tre staining in sputum and tongue swab-derived samples. Bright-green rods are considered positively stained. The magnification was 100x and scale bar represents 5 µm. Total number of bacilli scored for each sample type is shown in the top left corner and in **(B)**. In some cases, no positively-stained bacteria were detected. Numbers in top left corner represent the number of positively stained bacilli in each sample (refer to **Supplementary Figure 1** for representative images relative to four other random patient samples). **(B)** Quantitation of positively-stained bacilli graded according to zero, low (1-9), medium (10-99) and high (>100) in at least 100 fields of view. Table inset shows the predominant smear score (grey block) for the sample type (i.e. sputum or tongue swab) stained with either Auramine or DMN-Tre.

We next sought to establish whether using the viability stain, DMN-Trehalose (DMN-Tre), could improve the diagnostic utility of tongue swabs relative to the sputum. We simultaneously performed a matched auramine smear (independent of the data presented above) to interrogate AFB in each sample. If sputum samples were negative for AFB using auramine staining, it was assumed that DMN-Tre staining was negative due to the premise that viable organisms would only form a smaller proportion of the entire bacterial population. Based on these criteria, matched sputum and tongue swabs from only 28/89 (31%) participants were analyzed. For DMN-Tre staining, we designated any rod-shaped structure with the dimensions of 4 – 8 µm as positively stained (**Figure 3A** and **Supplementary Figures 1A–D** for representative images). The number of positively stained bacteria was determined using each stain and subsequently graded as ‘zero’ (0 bacilli), ‘low’ (1-9 bacilli), ‘medium’ (10-99 bacilli) and ‘high’(>100 bacilli). Auramine smears on sputum-derived samples appeared to be more sensitive with a higher frequency of samples containing ‘medium’ to ‘high’ bacterial loads. In contrast, auramine smears on tongue swabs either detected ‘zero’ or ‘low’ numbers of stained bacteria in at least 100 fields of view (**Figure 3B**). DMN-Tre staining on the corresponding sputum samples yielded lower positivity rates ranging from ‘zero’ to ‘medium’ whereas tongue swabs stained with DMN-Tre yielded either negative results or ‘low-grade’ positivity (**Figure 3B**).

### Relative quantification of DCTB captured by tongue swabs

**Figure 4:**
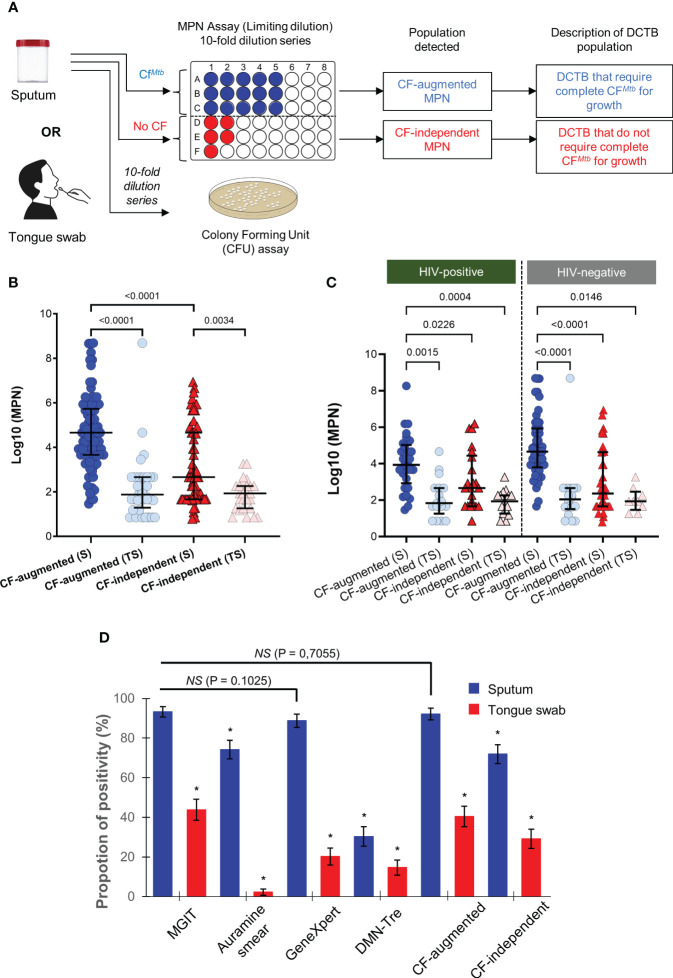
Use of DCTB assays and overall performance of sputum and tongue swabs for diagnostic testing. **(A)** Flow chart for assessment of DCTB in sputum and tongue swabs. Samples were decontaminated and the resulting bacteria enumerated by CFU/ml (for the conventionally culturable proportion) and Most Probable Number (MPN) limiting dilution assays containing CF with Rpfs (for the DCTB population). To control for the effect of CF in growth stimulation, un-supplemented media (no CF) was used as a control. The limiting dilution assays yield a MPN (see methods for further detail) which was then compared across categories. **(B)** Median MPN values for CF-augmented and CF-independent populations in sputum (S) or tongue swab (TS) samples. The majority of TS samples had zero CFU/ml. The graphs show MPN values for CF-augmented (blue) and CF–independent (red) populations. Error bars depict the interquartile range. A one-way ANOVA (Tukey’s multiple comparison test) was used for statistical testing. Only comparisons where P<0.005 are shown on the graph. Contaminated samples were not included in the analysis. **(C)** Median MPN values for CF-augmented (blue) and CF–independent (red) sputum (S) and tongue swabs (TS) stratified according to HIV-status. Statistical testing was performed using a one-way ANOVA (Tukey’s multiple comparison test) and only comparisons where P<0.005 are shown on the graph. **(D)** Overall performance of diagnostic utility for tests used. Positivity rate (%) for MGIT, Auramine smear, GeneXpert, MPN and DMN-Tre using sputum or tongue swab samples as a function of total number of participants (N = 89). As MGIT culture on sputum is considered the clinical standard, it was used as the reference sample for statistical testing. The McNemar test was thus used to calculate statistical significance between sputum and tongue swabs for each test, relative to the MGIT performed on sputum. P<0.05 was used as the cut-off for statistically significant differences. Significance is denoted as a * which represented P-values < 0.0001. No statistical significance was denoted by ‘NS’. All comparisons are shown in **Supplementary Table 1**.

After establishing that tongue swabs performed poorly relative to sputum on all routine clinical tests and viability staining, we next sought to investigate whether detecting DCTB in tongue swabs could improve diagnostic yield. DCTB are quantified using the most probable number (MPN) as obtained from the incidence of positive or negative growth in the limiting dilution assays shown in **Figure 4A**. CFUs are used as measure of plateable/culturable bacteria and to determine the quantum of DCTB by dividing log_10_(MPN) values by CFUs. In 25/89 (28%) sputum samples, no CFUs were obtained while 3/89 (3%) CFUs were contaminated. In tongue swabs, 82/89 (92%) samples yielded no CFUs while in 2/89 (2%), CFUs were contaminated. As a result, we opted to only report and analyze the log_10_(MPNs), and not DCTB, for every sample processed with or without CF supplementation (CF-augmented or CF-independent, respectively) to uncover organisms that failed to grow on solid media. Sputum samples were positive for CF-augmented MPNs in 82/89 (92%) (average log_10_(MPN) = 4.33; median log_10_(MPN) = 4.26) and 2/89 (2%) were contaminated. In contrast, tongue swabs were positive for CF-augmented MPNs in 36/89 (40%) samples (average log_10_(MPN) = 0.87 and median log_10_(MPN) = 0) and 1/89 (1%) was contaminated (**Figure 4B**; **Table 1**). This equated to approximately half (52%) (*P*<0.0001) the amount of CF-augmented DCTB retrieved using tongue swabs compared to sputum (**Figure 4B**). We simultaneously assessed the amount of CF-independent MPNs in both sample types using a media only control which served to identify organisms that spontaneously resumed growth in liquid media without the addition of CF supplementation. Overall, the amount of CF-independent MPNs was relatively higher in sputum with 64/89 (72%) positive samples and 1/89 (1%) contamination (average log_10_(MPN) = 2.26; median log_10_(MPN) = 1.66) compared to tongue swabs with 26/89 (29%) positive samples and 1/89 (1%) contamination (average log_10_(MPN) = 0.54; median log_10_(MPN) = 0) (*P*<0.005) (**Figure 4B**, **Table 1**). Taken together, these data confirmed that the quantum of non-culturable and viable bacteria captured on tongue swabs was much lower than sputum. Moreover, the poorer diagnostic utility of tongue swabs could not be attributed to the presence of non-culturable bacteria.

### Performance of tongue swabs in HIV infected participants

Given that the MGIT test displayed the highest sensitivity to detect *Mtb* in sputum (**Figure 2A**), it was used as the reference test for further analysis. We stratified test performance based on HIV status as HIV-*Mtb* coinfection is typically paucibacillary, adversely affecting diagnosis by conventional diagnostics, culture and/or smear microscopy (Montales et al., 2015). In total, there were 53 HIV-negative and 31 HIV-positive participants (CD4 range of 13 – 855 cells/mm^3^) in this cohort. The remaining 5 did not have their HIV status captured in the clinical files (**Figure 1**). In HIV-positive participants, sputum positivity for the MGIT, Auramine smear, GeneXpert, DMN-Tre, CF-augmented and CF-independent assays were 100, 61, 94, 26, 94 and 55%, respectively (**Supplementary Figure 2**). Positivity in tongue swabs from these participants were at 29, 0, 19, 13, 71 and 48%. In HIV-negative participants, sputum positivity for the MGIT, Auramine smear, GeneXpert, DMN-Tre, CF-augmented and CF-independent assays were 91, 81, 87, 30, 92 and 34%, respectively. Positivity in tongue swabs from these participants were at 49, 2, 17, 17, 72 and 17%, respectively (**Supplementary Figure 2**). Overall, MPN positivity in tongue swabs was consistently lower than sputum and did not appear to be influenced by HIV status (**Figure 4C**). However, detection of CF-augmented organisms from tongue swab was higher than when tested with the clinically standard MGIT assay, irrespective of HIV status, (*P*<0.001) (**Supplementary Figure 2**) suggesting that in combination with CF-supplementation, diagnostic yield may be improved.

### Overall performance of sputum and tongue swabs as a diagnostic tool for TB detection

In summary, we compared clinical and lab-based tests using sputum and tongue swab samples (**Figure 4D**). We represented overall positivity as a function of total number of participants. MGIT positivity on sputum derived samples was 92% whereas the corresponding tongue swabs dropped significantly to 44%. Auramine smear microscopy on sputum detected AFB in 74% of participants but this dropped in the corresponding tongue swabs (2%). The GeneXpert appeared to be less sensitive than the MGIT at 89 and 20% for sputum and tongue swabs, respectively The use of DMN-Tre did not significantly improve detection in either sputum or tongue swabs (34 and 17%, respectively). Finally, the use of MPN assays to detect DCTB showed that sputum samples were 92 and 72% positive for CF-augmented and CF-independent organisms, respectively. As with our other tests, tongue swabs showed a lower positivity for these organisms at 40 and 29%, respectively.

**Figure 5:**
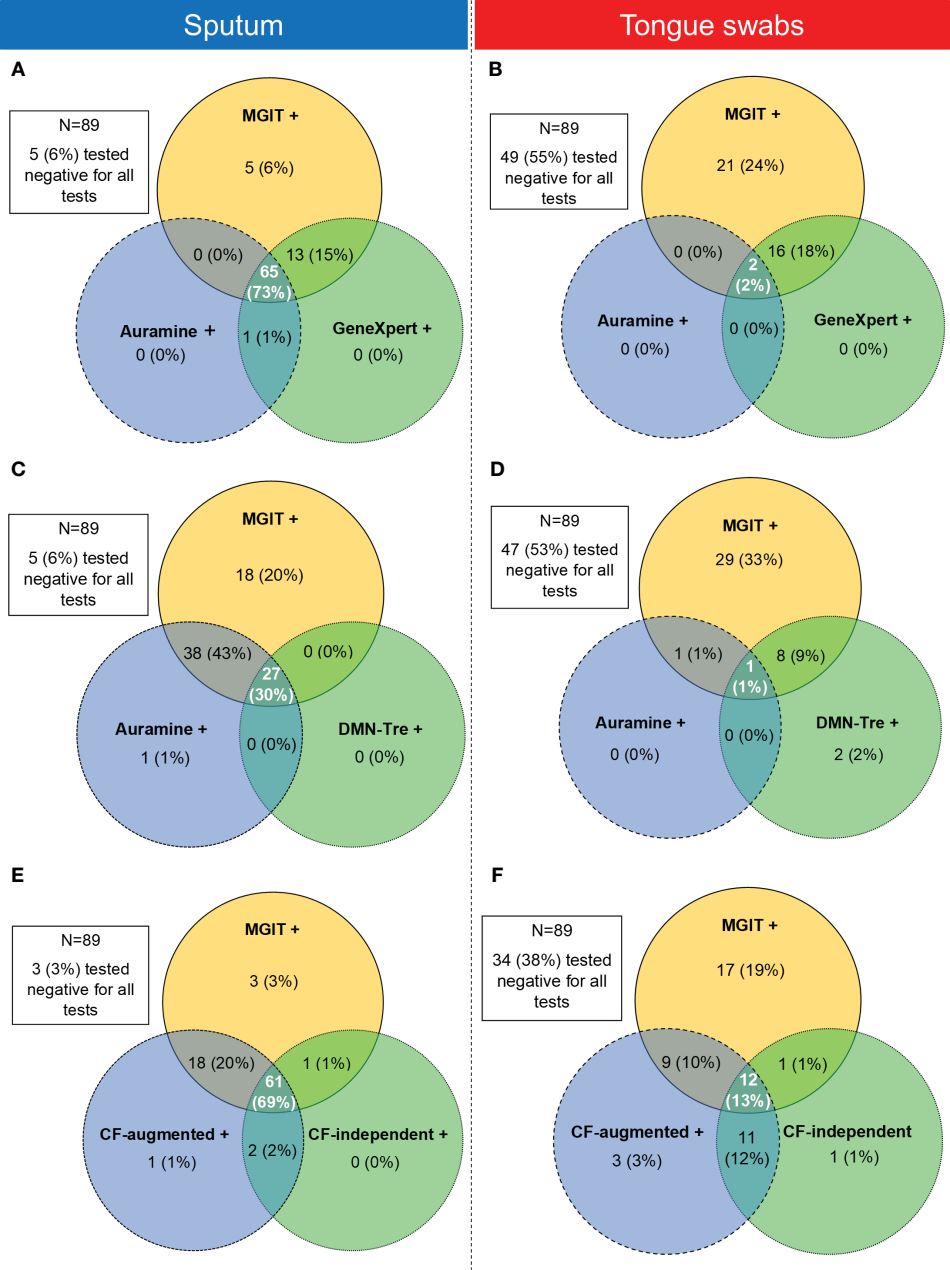
Venn diagrams showing the relationship between positivity rates between three tests simultaneously using sputum (left panel) or tongue swabs (right panel) as the testing sample. **(A, B)** Comparison between positive MGIT, Auramine smear and GeneXpert samples for sputum and tongue swabs, respectively. **(C, D)** Comparison between positive MGIT, Auramine smear and DMN-Tre samples for sputum and tongue swabs, respectively. **(E, F)** Comparison between positive MGIT, CF-augmented (CF-aug) and CF-independent (CF-ind) samples for sputum and tongue swabs, respectively. Intersecting areas show where positivity correlated between two or three tests simultaneously. Values in each circle represent where that test was positive while the other two were negative. The intersections between two circles represent where these two tests were simultaneously positive whilst the remaining test was negative. The middle intersection indicated where all three tests were positive simultaneously. Tests for all patients (N=89) were analyzed and where all three tests were simultaneously negative is indicated in the block outside of the Venn diagram.

**Figure 6:**
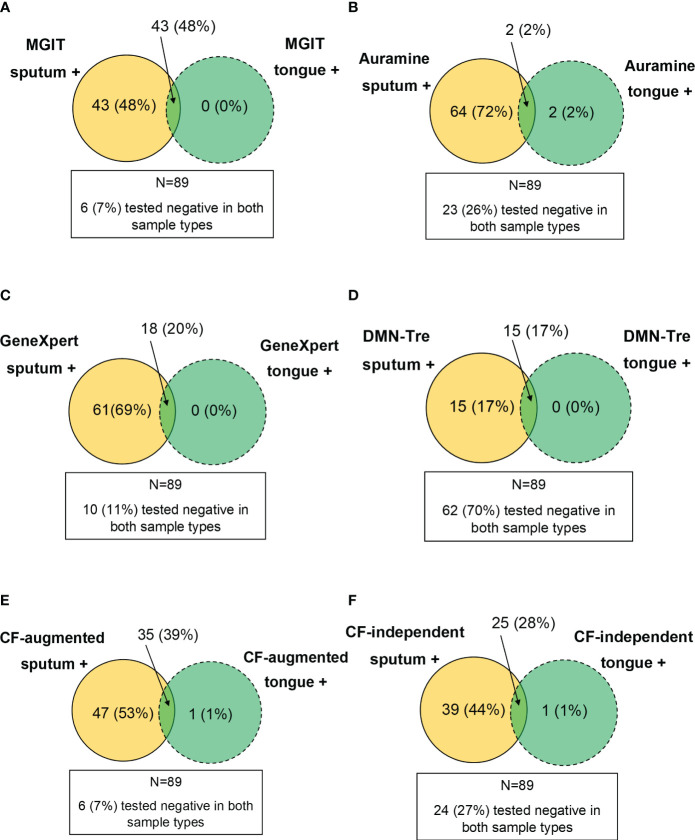
Performance of tongue swabs relative to sputum. Venn diagrams were constructed by assessing where a sample type was positive whilst the other was negative (i.e. value inside the circle). The intersection represents where both sample types were simultaneously positive. Results for all patients (N=89) were analyzed and where both sample types were simultaneously negative is indicated in the block outside of the Venn diagram. **(A–F)** represent MGIT, Auramine, GeneXpert, DMN-Tre, CF-augmented and CF-independent assays, respectively.

We also considered whether positivity could be described by taking into account which sample type detected *Mtb* simultaneously in all tests. These positive relationships between tests were represented using Venn diagrams. The MGIT sputum sample served as the comparator for all comparisons with sputum and tongue swab comparisons conducted separately (**Figure 5**). When comparing positivity between MGIT, GeneXpert and Auramine smear from sputum samples, 65/89 (73%) were simultaneously positive in all three (**Figure 5A**). For positivity between MGIT, Auramine smear and DMN-Tre, 27/89 (30%) were simultaneously positive in all three tests (**Figure 5C**). For positivity between MGIT, CF-augmented and CF-independent MPNs, 61/89 (69%) were simultaneously positive in all three tests (**Figure 5E**). Similar comparisons for tongue swabs revealed that for positivity between MGIT, GeneXpert and Auramine smear, 2/89 (2%) were simultaneously positive in all three (**Figure 5B**). For positivity between MGIT, Auramine smear and DMN-Tre, 1/89 (1%) was simultaneously positive in all three (**Figure 5D**). For positivity between MGIT, CF-augmented and CF-independent MPNs, 12/89 (13%) were simultaneously positive in all three (**Figure 5F**). The strength of agreement between the MGIT performed on sputum and all other tests was calculated and overall suggests that while tongue swabs are able to detect *Mtb*, positivity is consistently lower (**Supplementary Table 2**). We also compared the performance of each test between sample types. Generally, sputum samples were associated with a greater positivity rate relative to tongue swabs. In all cases, sputum detected all the positive cases identified by tongue swabs (**Figure 6**) unless there was a technical issue such as contamination in the culture-based assays (CF-augmented or CF-independent MPNs) (**Figures 6E, F**). This is likely reflective of the higher bacterial loads associated with sputum samples.

## Discussion

Despite specimen collection not always being easy or practical, sputum samples remain the standard for TB testing, particularly in high endemic settings (Mathebula et al., 2020). Recent studies suggest that tongue/oral swabs have utility for the detection of TB, but sensitivity appears to be variable and lower than for sputum on the GeneXpert or culture-based diagnostic tests (Wood et al., 2015; Luabeya et al., 2019; Nicol et al., 2019; Wood et al., 2021; Andama et al., 2022). This could be attributed to the GeneXpert being optimized for sputum testing only (Andama et al., 2022). Moreover, to achieve sensitivities comparable to sputum GeneXpert, multiple swabs could be combined before testing (Wood et al., 2015; Mesman et al., 2019; Nicol et al., 2019; Wood et al., 2021). We recently showed that tongue swabs could be used to detect TB in young, hospitalized children (Ealand et al., 2021). Other non-sputum specimens, such as urine, have also been assessed. Relative to confirmed culture and GeneXpert assays, the combined sensitivity and specificity of urine LAM and manual qPCR-based oral swab testing appeared to be significantly improved than the use of each non-sputum sample alone (Shapiro et al., 2022). It is accepted that oral and/or tongue swabs are smaller in volume and likely contain less *Mtb* bacilli resulting in differential diagnostic pickup. To address this, Andama et al. recently developed new pre-processing methods that produced higher diagnostic sensitivity (Andama et al., 2022). More recently, Cox et al. tested one or two oral swabs in children and concluded that the low sensitivity observed precluded using this approach for pulmonary TB confirmation due to poor yield (Cox et al., 2022b). The prevailing idea before starting our study was that tongue swabs and/or CF-supplementation might be able to identify paucibacillary TB (and mitigate false negatives) in patients that struggle to produce sputum. Here we chose to include TB-confirmed individuals, either *via* positive smears or GeneXpert, in order to assess the relative performance of tongue swabs against sputum. We were not able to say with certainty whether tongue swabs perform better in patients who can produce sputum and this therefore served as a proof of principle. In addition, we included HIV-positive patients which typically struggle to produce sputum and ran a suite of clinical tests on sputum and tongue swab samples.

Given that culture remains the gold standard for TB diagnosis, approaches to improving this with tongue swabs is paramount. Whilst being present in sputum (Chengalroyen et al., 2016; Gordhan et al., 2021; McIvor et al., 2021; Zainabadi et al., 2021; Zainabadi et al., 2022), it was unclear whether DCTB adversely affects diagnostic yield in tongue swabs. To address this, we assessed the diagnostic performance of tongue swabs relative to sputum using standard clinical tests, including MGIT, GeneXpert, Auramine smear microscopy and DCTB assays. We further assessed whether novel viability probes could be used to improve detection by smear microscopy (Kamariza et al., 2018). As expected, due to lower bacterial loads, MGIT time to positivity was significantly longer for tongue swab-derived specimens (Andama et al., 2022). The majority of sputum specimens (93%) were MGIT positive whereas only approximately half (44%) of the tongue swabs were positive, with a similar trend for GeneXpert. At the time of this study, the GeneXpert MTB/RIF assay was used which has a lower sensitivity compared to the GeneXpert MTB/RIF Ultra platform which, could have affected our results (Osei Sekyere et al., 2019).

Typically, smear-based detection of *Mtb*, such as Auramine, is associated with lower sensitivity in pulmonary and extra-pulmonary samples (Law et al., 2018; Arora and Dhanashree, 2020). This was corroborated in our study where smear microscopy on tongue swabs offered little diagnostic value as majority of the samples were either ‘negative’ or ‘scanty’. The use of DMN-Tre did not improve this outcome. We noted a lower sensitivity with DMN-Tre which could be due to several factors, including the requirement for washing steps during DMN-Tre staining; the non-specific staining of non-mycobacterial organisms in sputum; and no acid-washes to remove the non-specific straining. Alternatively, as Auramine detects both live and dead organisms, it may demonstrate better sensitivity than DMN-Tre which only detects live organisms. A limitation in our study was the assumption that viable *Mtb* organisms would only form a smaller proportion of the entire bacterial population as this influenced how we performed and analyzed smear microscopy. Newer fluorescent probes are currently being tested on sputum for improved sensitivity but have not yet been tested on tongue swabs (Kamariza et al., 2021).

Due to the poor performance of tongue swabs under routine diagnostic tests, we next sought to establish whether the lower sensitivities were associated with DCTB. In our cohort, CF-augmented and CF-independent DCTB were detected in approximately 90% and 70% of sputum samples, respectively. Compared to MGIT positivity on tongue swabs, detection of DCTB almost doubled in HIV-positive participants. The detection of more CF-augmented and CF-independent DCTB from tongue swabs, compared to MGIT, suggests that non-invasive sampling approaches, particularly in PLWH or children who struggle to produce sputum, holds great potential for TB diagnosis. Another limitation in our study was that a significant proportion of participants who might have been on anti-TB treatment for up to 5 days were enrolled. Whilst positivity was high, we cannot definitively say that some organisms were not killed by drug treatment. For those individuals who started treatment on the day of enrollment we anticipate a negligible effect. Given that TB clinically presents as a diverse spectrum ranging from latent to active disease, it is unlikely that a single sampling method can or should be applied for screening infected individual. Efforts to improve tongue/oral swab sensitivity have already shown promise and further testing combined with novel culture-based approaches should be prioritized in larger cohorts and clinical settings.

## Materials and methods

All methods were performed in accordance with the relevant regulations and guidelines for growth of *Mtb* and handling on human specimens and with approved clinical guidelines. All experiments involving live *Mtb* were conducted in a BioSafety Level III laboratory, registered with the South African Department of Agriculture Forestry and Fisheries (registration number: 39.2/NHLS-20/010).

### Study design and sample collection

Study participants were recruited prospectively from primary healthcare facilities in Soweto (Johannesburg, South Africa) through the clinical platforms of the Perinatal HIV Research Unit (PHRU). Enrollment criteria included written consent, older than 18 years of age, a positive GeneXpert or auramine smear. A tongue swab sample was collected before sputum collection by study site nurses. Institutionalized persons, multi-drug treatment for longer than 5 days or sputum samples received by the lab more than 4 hours after expectoration were excluded from this study. All Clinical tests were performed according to standard protocols and included MGIT assays (BACTEC™ MGIT 960), GeneXpert (Cepheid) and standard smear microscopy.

### Performance DMN-Trehalose relative to auramine staining

Following decontamination and de-clumping of sputum and tongue-swab samples, the bacteria were stained with Auramine or DMN-trehalose as described in our previous studies (Kamariza et al., 2018; Ealand et al., 2021). In the case of Auramine, stained samples were sealed using a glass cover-slip (22 × 40 mm) and nail-polish before viewing using a fluorescent microscope (Zeiss Observer Z1). The bacteria were viewed using two channels namely, DIC and FITC with exposure times of 100–150 and 3,000 ms, respectively. Spectral properties for FITC excitation and emission were 498 and 526 nm, respectively. In the case of DMN-Tre, 10 µl of each sample was aliquoted onto a 2% agarose pad and sealed with a cover slip (without nail-polish) (Skinner et al., 2013) for viewing in the DIC and FITC channels (Zeiss Observer Z1). Agarose pads promote bacterial setting on the same plane for better microscopy by virtue of the pores in the agarose. More bacteria, and other material, in the sample will be in focus when capturing images. The same parameters were used for both staining protocols and for each samples type.

### Most probable number (MPN) assay

The laboratory strain *Mtb* H37Rv was grown to logarithmic phase for the generation of culture filtrate (CF), for the detection of DCTB, as previously described (Chengalroyen et al., 2016). The liquid limiting dilution assay, hereafter referred to as the most probable number (MPN) assay was conducted as previously described (Chengalroyen et al., 2016). Briefly, CF derived from *Mtb* H37Rv was mixed in a 1:1 ratio with standard 7H9 media and PANTA antibiotic mixture (BD Biosciences). This was added in triplicate to one half of a 48-well microtiter plates (Thermo Scientific Nunc). A media only control containing PANTA antibiotic (i.e. no CF) was dispensed into the other half of the plate. Sputum (a random spot or overnight sample ranging between 2 – 5 ml) and tongue swab (placed into 3 ml transport media comprised of Middlebrook 7H9 media supplemented with OADC (BD) and Tween 80 (MerckSigma)) samples were decontaminated with an equal volume of *N*-acetyl-*L*-cysteine and sodium hydroxide (NaLc-NaOH) and finally resuspended in 2 ml 7H9 media (as above). Cells were then de-clumped by vortexing with 2 mm, sterile glass beads for three rounds of 10 s each. A 450 µl aliquot of each sample (sputum or tongue swab) was then added to the first well of a 48-well microtiter plate and serially diluted 10-fold until the end of the plate. In addition, select dilutions of the sputum and tongue swabs samples were plated on solid 7H11 media to determine the number of culturable bacteria (CFU/ml). After four to six weeks of incubation at 37°C, CFU/ml were counted and MPN plates were scored respectively, to determine the number of CF-augmented and -independent bacteria using online software (http://www.wiwiss.fu-berlin.de/fachbereich/vwl/iso/ehemalige/wilrich/index.html).

### Data analysis

GraphPad Prism (Version 9.5.1) for Windows (GraphPad Software, San Diego, California, USA) or Microsoft 365 Excel was used to generate all figures. All statistical analyses was performed using the appropriate tests in GraphPad Prism. For assessing the differences between MGIT time to positivity (TTP) and GeneXpert Ct values in sputum and tongue swab samples, paired t-tests were used. In both cases, since negative results were removed from the data analysis, instead of being captured as zero, the t-test only considered participants that had data in both specimen types. When comparing the log_10_(MPNs) recovered between sputum or tongue swab categories, i.e. CF-augmented or CF-independent, a one-way ANOVA combined with a Tukey’s multiple comparison test was used. When comparing proportions between tests from sputum and tongue swab samples, the non-parametric McNemar test (used to analyze paired data based on 2x2 contingency tables) was used to calculate statistical significance. Venn diagrams depicting the positivity rates between tests or sample types were constructed manually.

## Data availability statement

The original contributions presented in the study are included in the article/**Supplementary Material**. Further inquiries can be directed to the corresponding author.

## Ethics statement

The studies involving humans were approved by Human Research Ethics Committee of the University of the Witwatersrand. The studies were conducted in accordance with the local legislation and institutional requirements. The participants provided their written informed consent to participate in this study.

## Author contributions

BK conceived the overall concept of the study. CE, AS, JP, and BG executed the laboratory aspects of the study. CE and AS analyzed and curated data. CE wrote the first draft of the manuscript. NM and ZW recruited the study participants. CB and MK provided DMN-Trehalose for testing on clinical specimens. All authors contributed to the article and approved the submitted version.
