# Supplementary material for: The performance of tongue swabs for detection of pulmonary tuberculosis
Source: Front Cell Infect Microbiol. 2023 Sep 6;13:1186191. doi: 10.3389/fcimb.2023.1186191 (PMC10512057; doi:10.3389/fcimb.2023.1186191)
Supplement: Supplementary file 1 [file Presentation_1.pptx]

## Slide 1
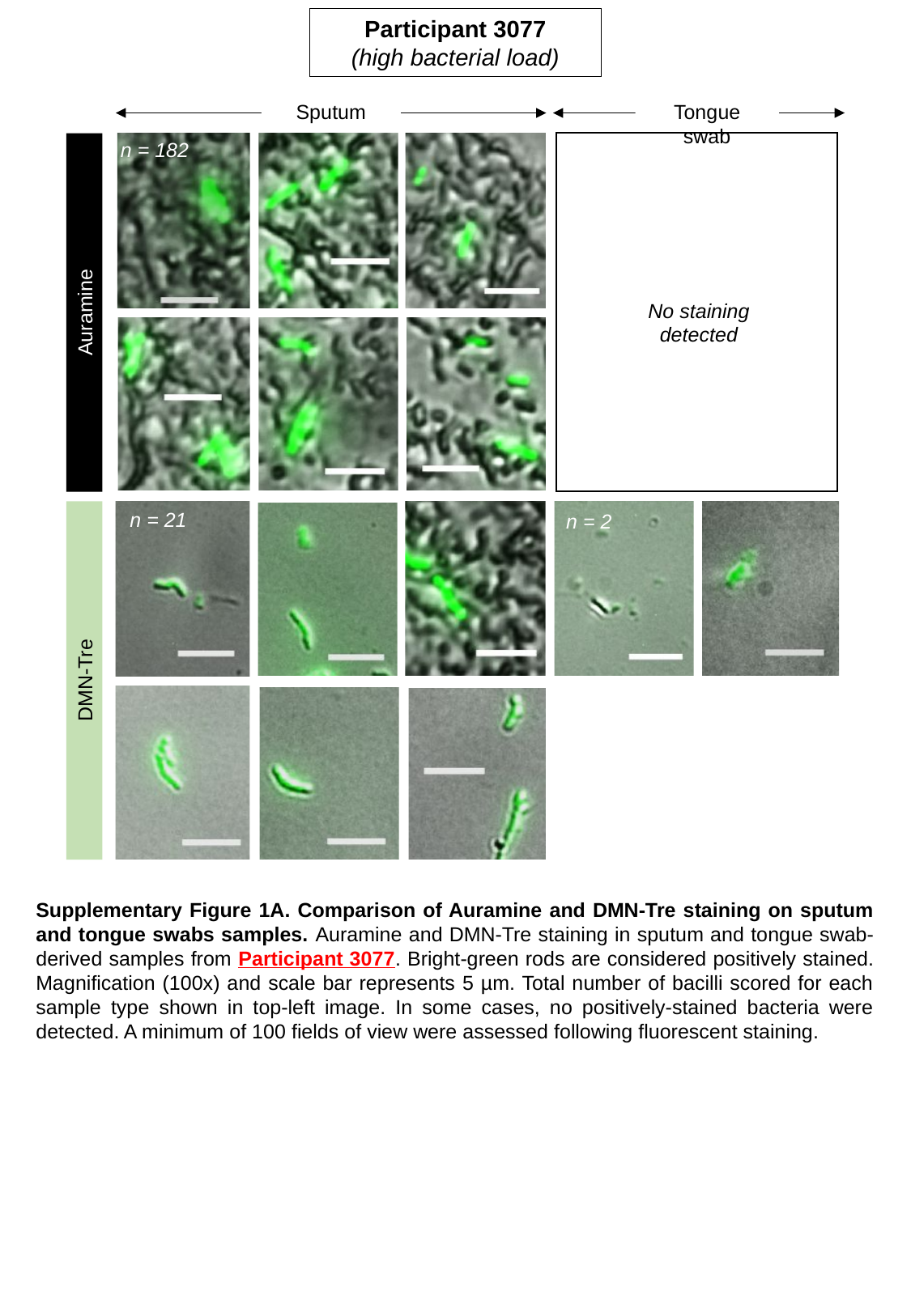

Participant 3077
(high bacterial load)
Sputum
Tongue swab
n = 182
Auramine
n = 21
n = 2
DMN-Tre
No staining detected
Supplementary Figure 1A. Comparison of Auramine and DMN-Tre staining on sputum and tongue swabs samples. Auramine and DMN-Tre staining in sputum and tongue swab-derived samples from Participant 3077. Bright-green rods are considered positively stained. Magnification (100x) and scale bar represents 5 µm. Total number of bacilli scored for each sample type shown in top-left image. In some cases, no positively-stained bacteria were detected. A minimum of 100 fields of view were assessed following fluorescent staining.

## Slide 2
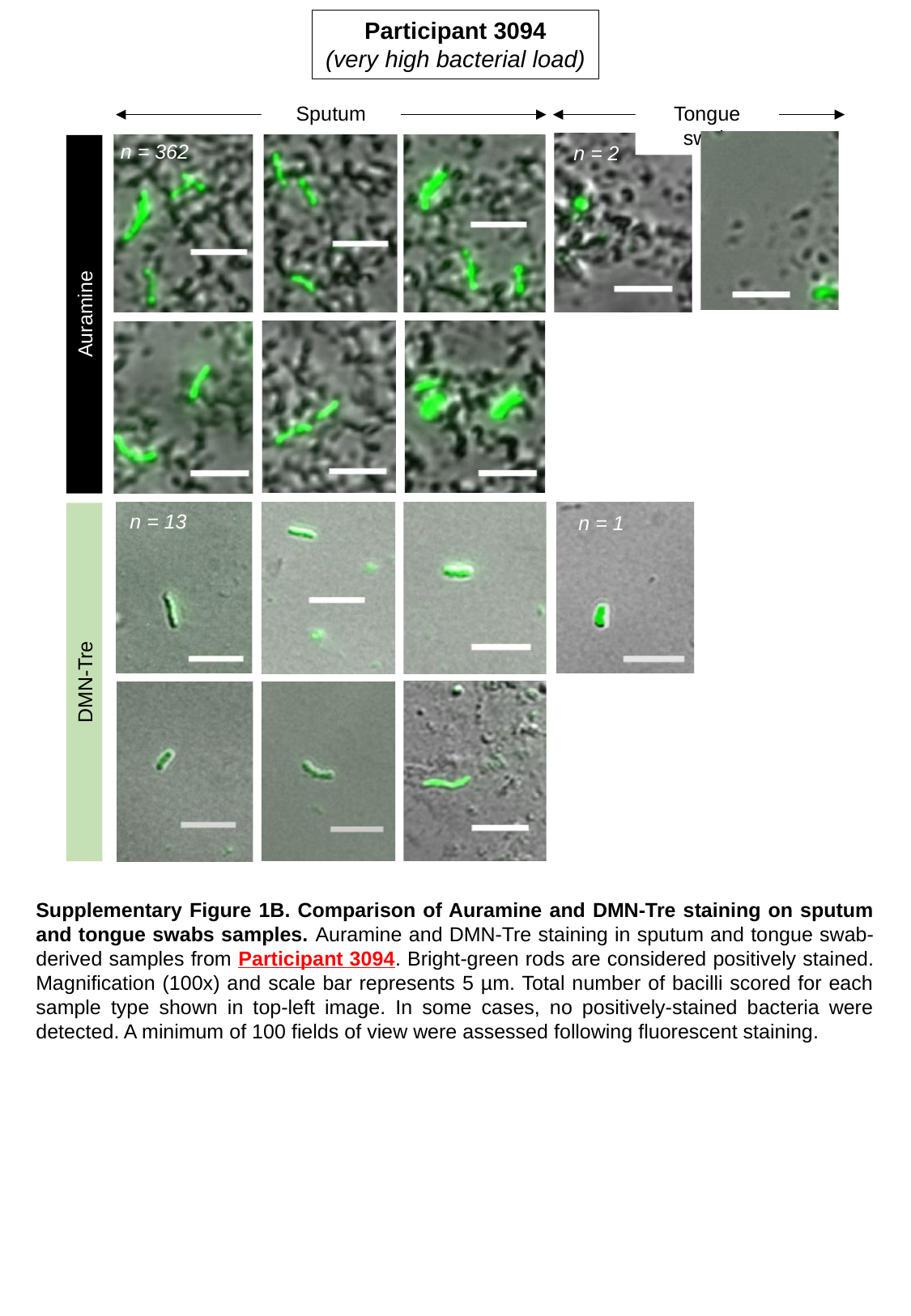

Participant 3094
(very high bacterial load)
Sputum
Tongue swab
n = 362
n = 2
Auramine
n = 13
n = 1
DMN-Tre
Supplementary Figure 1B. Comparison of Auramine and DMN-Tre staining on sputum and tongue swabs samples. Auramine and DMN-Tre staining in sputum and tongue swab-derived samples from Participant 3094. Bright-green rods are considered positively stained. Magnification (100x) and scale bar represents 5 µm. Total number of bacilli scored for each sample type shown in top-left image. In some cases, no positively-stained bacteria were detected. A minimum of 100 fields of view were assessed following fluorescent staining.

## Slide 3
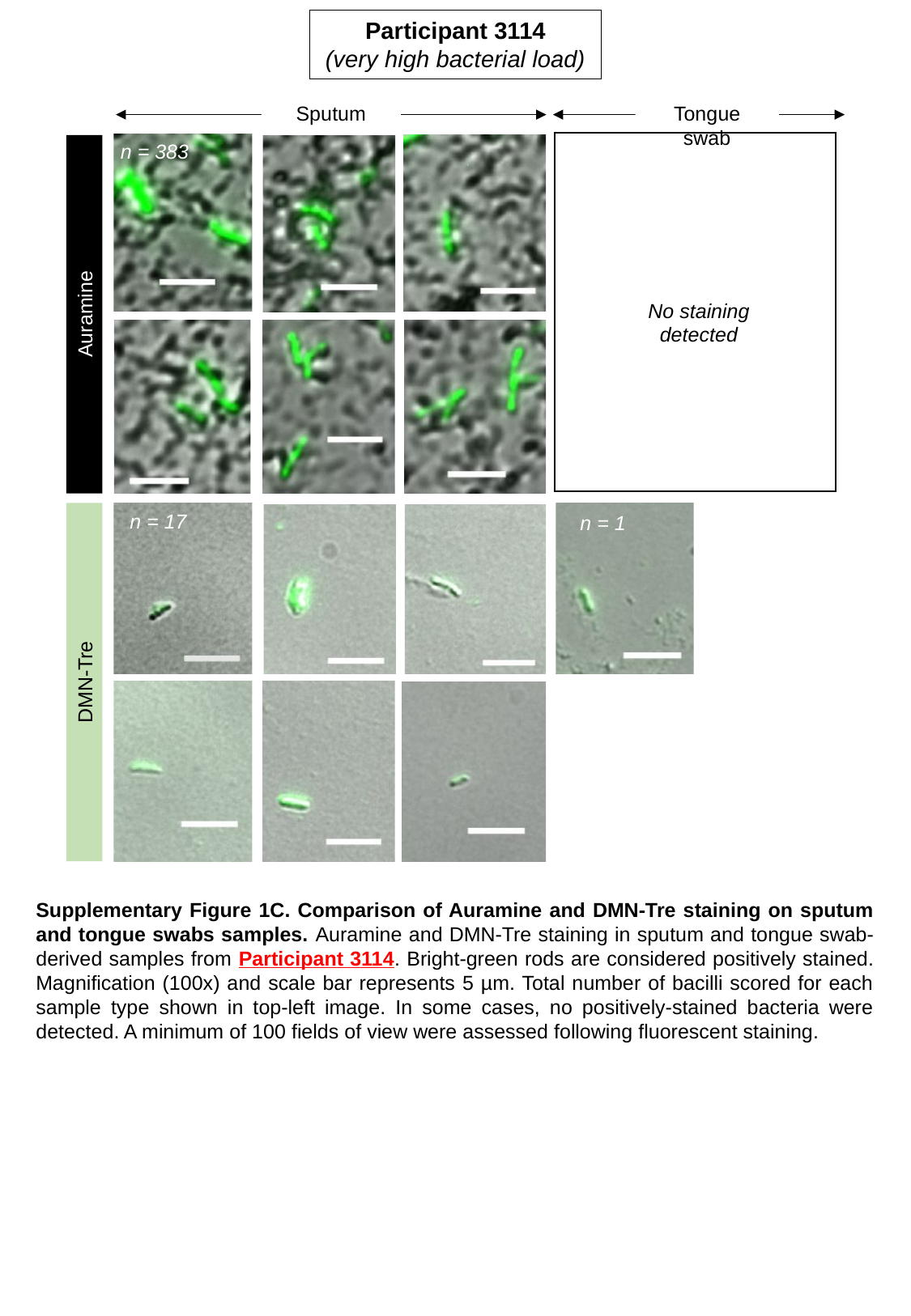

Participant 3114
(very high bacterial load)
Sputum
Tongue swab
n = 383
Auramine
n = 17
n = 1
DMN-Tre
No staining detected
Supplementary Figure 1C. Comparison of Auramine and DMN-Tre staining on sputum and tongue swabs samples. Auramine and DMN-Tre staining in sputum and tongue swab-derived samples from Participant 3114. Bright-green rods are considered positively stained. Magnification (100x) and scale bar represents 5 µm. Total number of bacilli scored for each sample type shown in top-left image. In some cases, no positively-stained bacteria were detected. A minimum of 100 fields of view were assessed following fluorescent staining.

## Slide 4
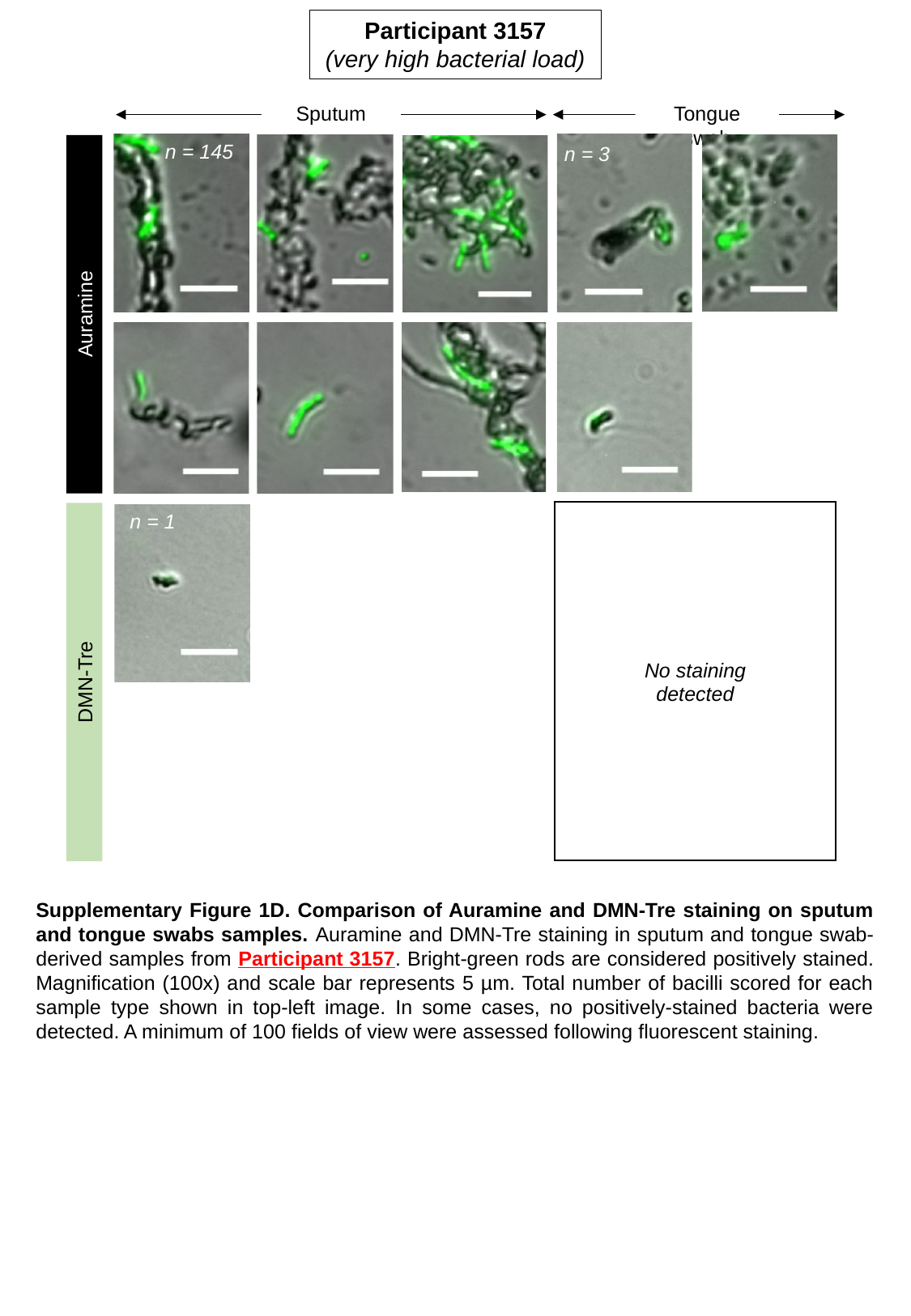

Participant 3157
(very high bacterial load)
Sputum
Tongue swab
n = 145
n = 3
Auramine
n = 1
n = 0
No staining detected
DMN-Tre
Supplementary Figure 1D. Comparison of Auramine and DMN-Tre staining on sputum and tongue swabs samples. Auramine and DMN-Tre staining in sputum and tongue swab-derived samples from Participant 3157. Bright-green rods are considered positively stained. Magnification (100x) and scale bar represents 5 µm. Total number of bacilli scored for each sample type shown in top-left image. In some cases, no positively-stained bacteria were detected. A minimum of 100 fields of view were assessed following fluorescent staining.

## Slide 5
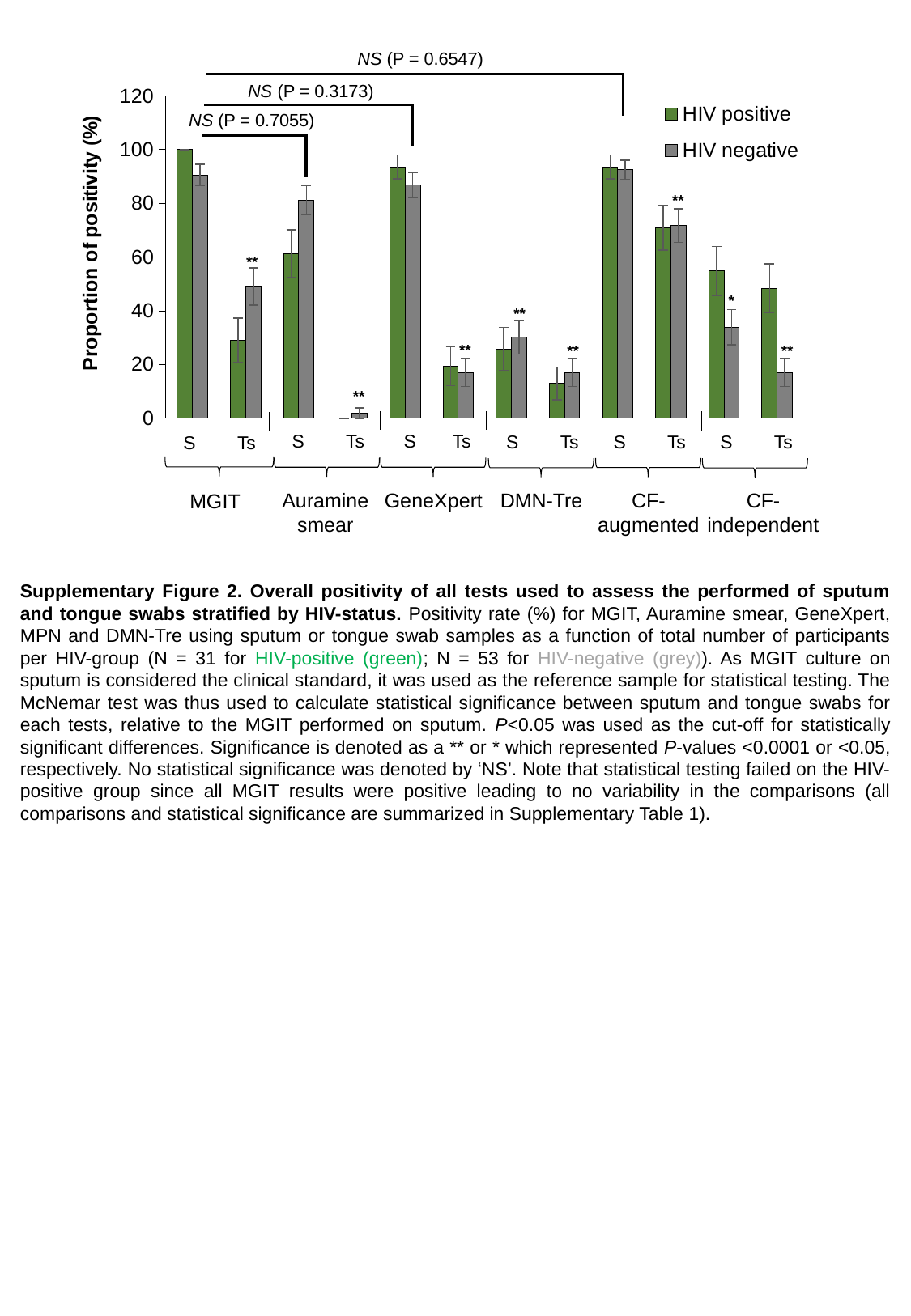

NS (P = 0.6547)
### Chart
| Category | HIV positive | HIV negative |
|---|---|---|
| MGIT (S) | 100.0 | 90.57 |
| MGIT (TS) | 29.03 | 49.059999999999995 |
| Auramine (S) | 61.29 | 81.13 |
| Auramine (TS) | 0.0 | 1.8870000000000002 |
| GeneXpert (S) | 93.55 | 86.79 |
| GeneXpert (TS) | 19.35 | 16.98 |
| DMN-Tre (S) | 25.81 | 30.19 |
| DMN-Tre (TS) | 12.9 | 16.98 |
| CF-dependent (S) | 93.55 | 92.45 |
| CF-dependent (TS) | 70.97 | 71.7 |
| CF-independent (S) | 54.84 | 33.96 |
| CF-independent (TS) | 48.39 | 16.98 |
 S Ts
Auramine smear
 S Ts
GeneXpert
 S Ts
CF-augmented
 S Ts
DMN-Tre
 S Ts
MGIT
 S Ts
CF-independent
NS (P = 0.3173)
NS (P = 0.7055)
**
**
*
**
**
**
**
**
Supplementary Figure 2. Overall positivity of all tests used to assess the performed of sputum and tongue swabs stratified by HIV-status. Positivity rate (%) for MGIT, Auramine smear, GeneXpert, MPN and DMN-Tre using sputum or tongue swab samples as a function of total number of participants per HIV-group (N = 31 for HIV-positive (green); N = 53 for HIV-negative (grey)). As MGIT culture on sputum is considered the clinical standard, it was used as the reference sample for statistical testing. The McNemar test was thus used to calculate statistical significance between sputum and tongue swabs for each tests, relative to the MGIT performed on sputum. P<0.05 was used as the cut-off for statistically significant differences. Significance is denoted as a ** or * which represented P-values <0.0001 or <0.05, respectively. No statistical significance was denoted by ‘NS’. Note that statistical testing failed on the HIV-positive group since all MGIT results were positive leading to no variability in the comparisons (all comparisons and statistical significance are summarized in Supplementary Table 1).
