# Supplementary material for: The performance of tongue swabs for detection of pulmonary tuberculosis
Source: Front Cell Infect Microbiol. 2023 Sep 6;13:1186191. doi: 10.3389/fcimb.2023.1186191 (PMC10512057; doi:10.3389/fcimb.2023.1186191)
Supplement: Supplementary file 2 [file DataSheet_1.docx]

**Supplementary Table 1.** McNemar statistical testing to detect significant differences between all tests relative to MGIT performed on sputum based on total cohort (N=89) or HIV-status (N=53 and N=31 for HIV-negative and HIV-positive, respectively).

| **Overall positivity** | |
| --- | --- |
| **Comparison** | **McNemar *P*-value** |
| MGIT_sputum vs. MGIT_Tongue | <.0001 |
| MGIT_sputum vs. Auramine_Sputum | <.0001 |
| MGIT_sputum vs. Auramine_Tongue | <.0001 |
| MGIT_sputum vs. GX_Sputum | 0.1025 |
| MGIT_sputum vs. GX_Tongue | <.0001 |
| MGIT_sputum vs. CF_dependent_Sputum | 0.7055 |
| MGIT_sputum vs. CF_independent_Sputum | <.0001 |
| MGIT_sputum vs. CF_dependent_Tongue | <.0001 |
| MGIT_sputum vs. CF_independent_Tongue | <.0001 |
| MGIT_sputum vs. DMN-Tre_Sputum | <.0001 |
| MGIT_sputum vs. DMN-Tre_Tongue | <.0001 |
|  |  |
| **HIV-negative** | |
| **Comparison** | **McNemar *P*-value** |
| MGIT_sputum vs. MGIT_tongue | <.0001 |
| MGIT_sputum vs. Smear_Sputum | 0.0588 |
| MGIT_sputum vs. Smear_Tongue | <.0001 |
| MGIT_sputum vs. GX_Sputum | 0.3173 |
| MGIT_sputum vs. GX_Tongue | <.0001 |
| MGIT_sputum vs. CF_dependent_Sputum | 0.6547 |
| MGIT_sputum vs. CF_independent_Sputum | 0.0075 |
| MGIT_sputum vs. CF_dependent_Tongue | <.0001 |
| MGIT_sputum vs. CF_independent_Tongue | <.0001 |
| MGIT_sputum vs. DMN_Sputum | <.0001 |
| MGIT_sputum vs. DMN_Tongue | <.0001 |
|  |  |
| **HIV-positive** | |
| Analysis fails because all MGITS are Positive thus no variability in the comparisons | |

**Supplementary Table 2.** Strength of agreement between MGIT sputum results and other diagnostic biomarkers (sputum and tongue swabs).

| **Variable** | **Overall** | **TB Confirmed** | **Positive** | **Kappa statistic ^#^** |
| --- | --- | --- | --- | --- |
|  |  |  |  |  |
| **MGIT (tongue)** |  |  |  |  |
| Negative | 50/89 (56.18) | 6/6 (100.0) | 44/83 (53.01) | 0.1068 |
| Positive | 39/89 (43.82) | 0/6 (0.00) | 39/83 (46.99) |  |
|  |  |  |  |  |
| **Auramine Smear (sputum)** |  |  |  |  |
| Negative | 23/89 (25.84) | 5/6 (83.33) | 18/83 (21.69) | 0.2664 |
| Positive | 66/89 (74.16) | 1/6 (16.67) | 65/83 (78.31) |  |
|  |  |  |  |  |
| **Auramine Smear (tongue)** |  |  |  |  |
| Negative | 87/89 (97.75) | 6/6 (100.0) | 81/83 (97.59) | 0.0033 |
| Positive | 2/89 (2.25) | 0/6 (0.00) | 2/83 (2.41) |  |
|  |  |  |  |  |
| **GeneXpert (sputum)** |  |  |  |  |
| Negative | 10/89 (11.24) | 5/6 (83.33) | 5/83 (6.02) | 0.5905 |
| Positive | 79/89 (88.76) | 1/6 (16.67) | 78/83 (93.98) |  |
|  |  |  |  |  |
| **GeneXpert (tongue)** |  |  |  |  |
| Negative | 71/89 (79.78) | 6/6 (100.0) | 65/83 (78.31) | 0.0360 |
| Positive | 18/89 (20.22) | 0/6 (0.00) | 18/83 (21.69) |  |
|  |  |  |  |  |
| **DMN-Tre (sputum)** |  |  |  |  |
| Negative | 62/89 (69.66) | 6/6 (100.0) | 56/83 (67.47) | 0.0610 |
| Positive | 27/89 (30.34) | 0/6 (0.00) | 27/83 (32.53) |  |
|  |  |  |  |  |
| **DMN-Tre (tongue)** |  |  |  |  |
| Negative | 76/89 (85.39) | 6/6 (100.0) | 70/83 (84.34) | 0.0244 |
| Positive | 13/89 (14.61) | 0/6 (0.00) | 13/83 (15.66) |  |
|  |  |  |  |  |
| **CF-dependent (sputum)** |  |  |  |  |
| Negative | 7/89 (7.87) | 3/6 (50.00) | 4/83 (4.82) | 0.4194 |
| Positive | 82/89 (92.13) | 3/6 (50.00) | 79/83 (95.18) |  |
|  |  |  |  |  |
| **CF-dependent (tongue)** |  |  |  |  |
| Negative | 53/89 (59.55) | 3/6 (50.00) | 50/83 (60.24) | -0.0221 |
| Positive | 36/89 (40.45) | 3/6 (50.00) | 33/83 (39.76) |  |
|  |  |  |  |  |
| **CF-independent (sputum)** |  |  |  |  |
| Negative | 25/89 (28.09) | 4/6 (66.67) | 21/83 (25.30) | 0.1675 |
| Positive | 64/89 (71.91) | 2/6 (33.33) | 62/83 (74.70) |  |
|  |  |  |  |  |
| **CF-independent (tongue)** |  |  |  |  |
| Negative | 63/89 (70.79) | 5/6 (83.33) | 58/83 (69.88) | 0.0249 |
| Positive | 26/89 (29.21) | 1/6 (16.67) | 25/83 (30.12) |  |
|  |  |  |  |  |

# **Kappa** ranges in value from 0 to 1 with a value of 1 meaning perfect agreement (negative values are possible.) The higher the value of Kappa, the better the strength of agreement. Strength of agreement: < 0.00 = poor; 0.00-0.20 = slight; 0.20-0.40 = fair; 0.41-0.60 = moderate; 0.61-0.80 = substantial; 0.81-1.00 = almost perfect.
